# Improving disease prevention, diagnosis, and treatment using novel bionic technologies

**DOI:** 10.1002/btm2.10359

**Published:** 2022-06-21

**Authors:** Albert Manero, Kaitlyn E. Crawford, Hannah Prock‐Gibbs, Neel Shah, Deep Gandhi, Melanie J. Coathup

**Affiliations:** ^1^ Limbitless Solutions University of Central Florida Orlando Florida USA; ^2^ Biionix Cluster University of Central Florida Orlando Florida USA; ^3^ Department of Materials Science and Engineering University of Central Florida Orlando Florida USA; ^4^ College of Medicine University of Central Florida Orlando Florida USA

**Keywords:** bioelectronics, biointerfaces, materials, medical devices, tissue repair and regeneration, wearables

## Abstract

Increased human life expectancy, due in part to improvements in infant and childhood survival, more active lifestyles, in combination with higher patient expectations for better health outcomes, is leading to an extensive change in the number, type and manner in which health conditions are treated. Over the next decades as the global population rapidly progresses toward a super‐aging society, meeting the long‐term quality of care needs is forecast to present a major healthcare challenge. The goal is to ensure longer periods of good health, a sustained sense of well‐being, with extended periods of activity, social engagement, and productivity. To accomplish these goals, multifunctionalized interfaces are an indispensable component of next generation medical technologies. The development of more sophisticated materials and devices as well as an improved understanding of human disease is forecast to revolutionize the diagnosis and treatment of conditions ranging from osteoarthritis to Alzheimer's disease and will impact disease prevention. This review examines emerging cutting‐edge bionic materials, devices and technologies developed to advance disease prevention, and medical care and treatment in our elderly population including developments in smart bandages, cochlear implants, and the increasing role of artificial intelligence and nanorobotics in medicine.

## 1 INTRODUCTION

The remarkable increase in average life expectancy during the 20th century ranks as one of society's greatest achievements.^1^ Although most babies born in 1900 did not live past age 50, babies born today in Japan, where life expectancy is the longest, might expect to live beyond 83 years. Life expectancy has now increased to at least 81 years in several other countries.^2^ As a result, the world's elderly population (aged ≥65 years) continues to grow at an unprecedented rate. An aging society is defined as a society with more than 7% of the population aged 65 or older; a population with more than 14% is an aged society, and a population with more than 20% is defined as a super aging society.^3^ Record life expectancy does not appear to be approaching its limit. Today, 8.5% of people worldwide (617 million) are elderly and according to a report in 2016, this percentage is projected to rise to nearly 17% by 2050 (1.6 billion).^4^ The global number of centenarians is expected to increase 10‐fold between 2010 and 2050^2^ and we will soon have a greater number of elderly people than children under the age of 5.^1^, ^5^ That we are living longer is clear, the important question is, are we living better?^1^ As the proportion of the oldest‐old (aged >85 years) and length of life expectancy increases, a rise in age‐related degenerating diseases, disability, and prolonged dependency is projected.^6^, ^7^ Indeed, this predicts that reductions in mortality may well be accompanied by increases in chronic disease. For example, a rise in the prevalence of chronic diseases including heart disease, arthritis, and diabetes was recorded in elderly people in the United States,^8^ Netherlands,^9^ and Sweden.^10^ Increases in musculoskeletal pain, dizziness, leg ulcers, hypertension^9^, ^11^ and worsening lung function^12^ have also been reported in the population aged 77 years and over. Further, an increase in disease and chronic conditions has been reported in the youngest‐old people aged 65–69 years.^13^ The apparent contradiction in improved survival and increased chronic disease is at least partly accounted for by early diagnosis, improved treatments, and the prevalence of chronic conditions that can now be medically managed.^1^, ^14^ For example, initially silent diseases, such as type 2 diabetes, hypertension, and some cancers, now get diagnosed earlier and patients are treated with more efficacious, contemporary therapeutics.^15^, ^16^ These advancements correlate with a longer period of morbidity, but with an improved functional status and potentially less severe disabilities in activities of daily living (e.g., feeding, dressing, bathing, or showering).

**FIGURE 1:**
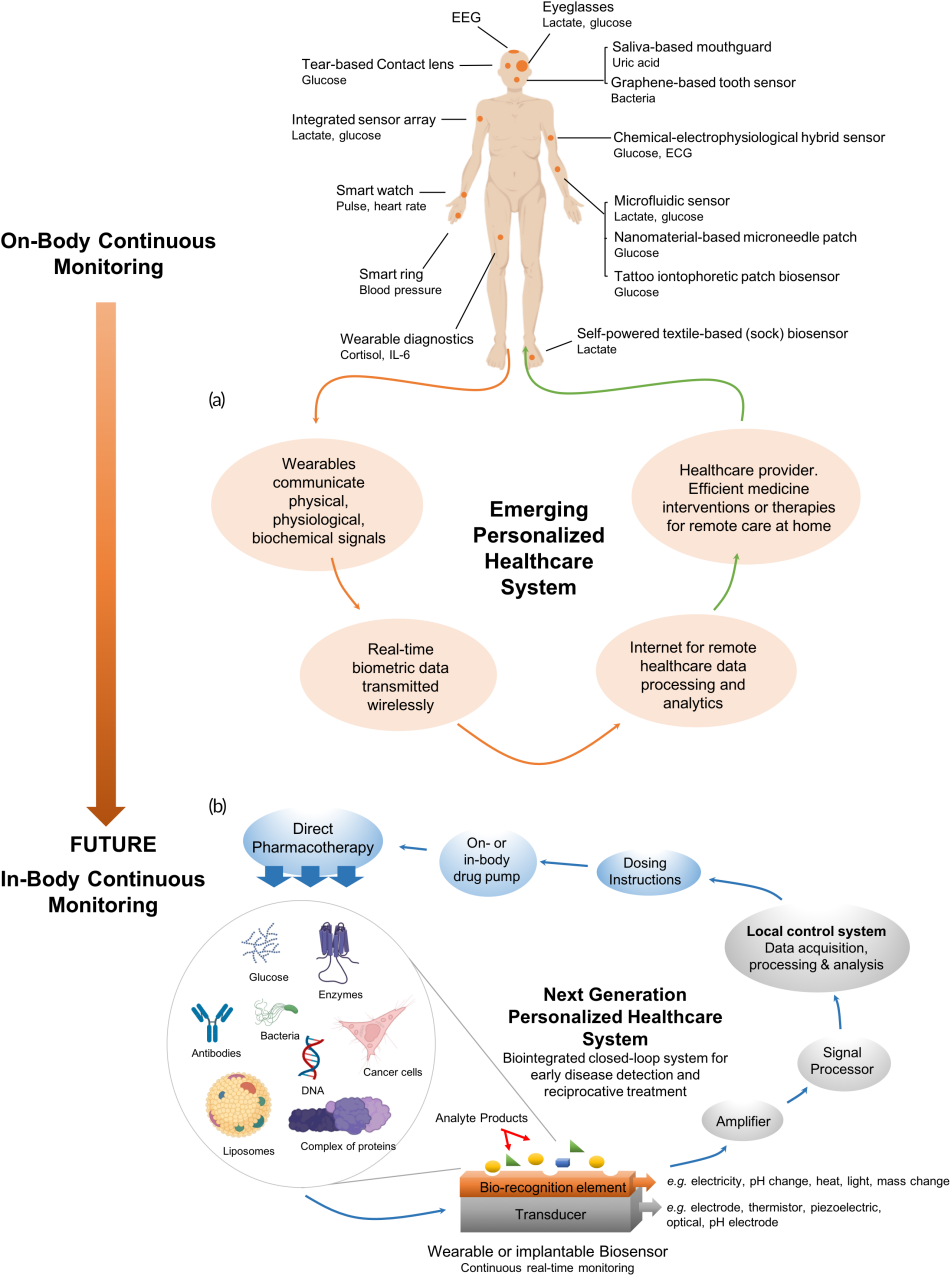
Advanced wearable electrochemical transducer platforms offer many advantages as wearable sensors for physiological monitoring and are easily integrated onto textile materials or directly on the skin, making this a rapidly growing market with a US$13 billion annual turnover.^18^ (a) On‐body continuous monitoring aims to transform centralized hospital‐based care systems to home‐based personal medicine, reducing healthcare cost and time for diagnosis. Despite significant advances in biosensor technology, successful commercialized devices remain few, although the glucose sensors are an exception. (b) Next generation on‐ and in‐body biosensors are forecast to be responsive systems where a target substrate/s (e.g., antibody, protein, DNA) is detected by a bio‐recognition system. Substrate interaction, leads to a signal, for example, an electrical current, pH change, chemical, gas, light, heat or mass change. The transducer converts this into a conventional electrical signal, which is amplified and the data collected are used to initiate a reciprocal response, such as to instruct an on‐ or in‐body drug pump to deliver a specified dose to the patient. This closed‐loop system could offer superior therapeutic activity and play a critical role in early disease detection.

**FIGURE 2:**
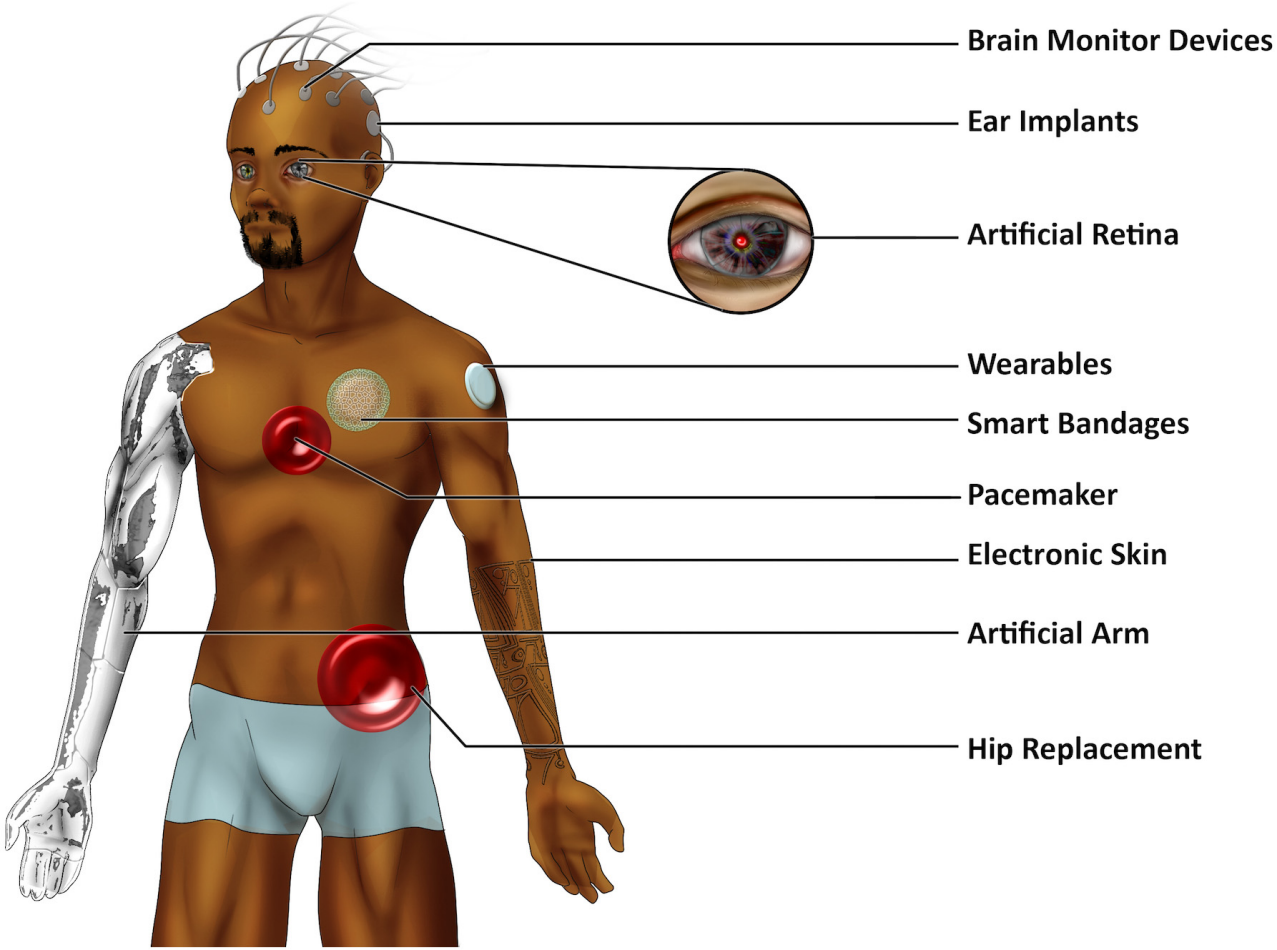
A schematic image demonstrating examples of both implantable and wearable, permanent and temporary medical technologies including, cochlear implants, brain‐machine technology, the artificial retina, electronic skin, neuro‐prosthetics, orthopedic implants and smart bandages

Increasing numbers of people at old and very old ages will pose major challenges for health‐care systems. Consequently, scientific innovation and public and private investments in biotechnology have caused the Bio‐Implants market to flourish (10.1% Compound Annual Growth Rate [CAGR] with a forecast of >US $134 billion by 2024). Discoveries in both implantable and wearable devices are meant to ensure longer periods of good health, a sustained sense of well‐being, and extended periods of activity, social engagement, and productivity.^17^ Advancements in science and engineering have allowed us to enter a new phase in both our understanding and use of translational materials in medicine. The ultimate goal in bionic materials and device therapy is to innovate and develop new and improved technology to diagnose and treat age‐related damage to human tissue, thereby preventing or postponing age‐associated disease. During the early stage of many diseases, subtle and often nondescript physicochemical changes can occur prior to the onset of an illness and before manifesting physically. Wireless technologies capable of detecting and treating the earliest signs of disease are fast becoming the diagnosticians front‐line defense. Other medical devices may also provide a more effective means of self‐management for elderly patients with chronic disease, undoubtedly improving their quality of life (Figure 1). These prospects provide groundwork that inspires the design of diverse technologies ranging from hybrid inorganic–organic devices to electro‐active medical implants. Designing responsive systems that can monitor electrical and/or biochemical on‐body signals, continuously and in real‐time, may transform centralized hospital‐based care systems to home‐based personal medicine, reducing healthcare costs and time for diagnosis. These advancements would enable practitioners to provide more informed interventions at time points that have the potential to be life changing and life saving. Furthermore, on‐ and in‐body systems that are able to respond to these physiochemical changes by immediately stimulating the targeted organ or tissue, could offer superior therapeutic activity, and play a critical role in future clinical medicine. As such, the development of biointegrated implants that combine a closed‐looped system and on demand, continuous real‐time monitoring have been identified as the next generation of implants.^17^, ^18^ To effectively achieve its goal, biointerfaces capable of assessing anatomy, morphology, functional and molecular changes, and cellular responses to surface parameters such as design, geometry, chemistry, scaffold release kinetics, and changes in structural and mechanical behavior are required. Therefore, smart macro, micro, and nanoscale strategies require the use of versatile and sophisticated techniques and technology. Newer biomaterials that are chemoresponsive, electro‐active, photomechanical, piezoelectric, temperature responsive, thermoelectric, pH‐sensitive, or contain shape‐memory behavior will undoubtedly contribute to novel technological endeavors. Fundamentally, many contemporary technological discoveries focus on both permanent and temporary medical solutions to be worn, implanted or ingested, that replace, repair or even augment human performance and intelligence beyond their understood normal limits. Emerging areas include ophthalmic and neurostimulator implants, dental, orthopedic and trauma implants, spinal implants, cardiovascular implants, pacing devices, stents as well as artificial organs and in tissue repair (Figure 2). Interestingly, increasing awareness of new technology is also causing a rise in the number of people not only opting for surgery, but with many new patient‐customers seeking preventative ahead of curative electronic solutions.^4^ This review examines cutting‐edge “bionic” materials, devices, and technologies developed to advance medical diagnoses and treatments. This review is not intended to be exhaustive, but rather a summary that highlights recent progress in areas focusing on solving specific unmet clinical needs within our growing elderly population. Here, we highlight advancements in four areas of research: (1) Wearable smart bandage technologies designed to target challenges associated with chronic wound healing. (2) The neuroprosthetic implantable cochlear device developed to ameliorate hearing loss. (3) Artificial intelligence (AI) as it relates to reshaping medical care services for older adults. And, (4) bioinspired micron and nanorobotic technologies able to intervene in biological processes at the molecular level, offering the promise of major future advances in medical diagnosis and treatment for the elderly.

## 2 THE SMART BANDAGE

Chronic wounds are estimated to affect 2.4–4.5 million people in the United States at an economic cost of $20 billion per year.^19^, ^20^ Wound care in adults ≥65 years accounts for the majority of these costs^21^ and this economic burden is projected to further increase due to an aging population and greater incidence of comorbidities such as diabetes. Chronic wounds including venous leg ulcers, diabetic foot ulcers, arterial insufficiency, and pressure ulcers disproportionately affect the elderly and impose substantial morbidity and mortality on millions of older Americans.^21^ Remarkably, the incidence of venous leg ulcers is presently three to four times higher and pressure ulcers five to seven times higher in persons aged ≥80 years when compared with those aged 65–70 years.^22^, ^23^ Approximately 20% of adults aged ≥65 years have a known diagnosis of diabetes while a further 16% are estimated to be unaware of being diabetic.^24^ Between 1997 and 2010, the prevalence of diabetes in the elderly increased by 62% making it a significant and growing public health problem. Insulin resistance is associated with advancing age and is reported to be due to a combination of adiposity, sarcopenia, physical inactivity and a decline in pancreatic islet function and proliferative capacity.^25^, ^26^ Underlying health conditions such as diabetes can overwhelm the regenerative capacity of skin and impair the healthy healing response.^27^ As a result, 90% of wound ulcers that are slow to heal become infected^28^ and lack of a competent immune response can lead to bacteremia and sepsis.^29^ The infection may further halt the healing process and presently chronic wounds lead to loss of limb function and are also one of the predominant causes of nontraumatic limb amputations worldwide.^20^ Wound healing technologies for both functional and cosmetic improvements constitute a major medical commercial enterprise, with the market for wound closure and scar‐free healing products^30^ exceeding $15 billion and the market for skin scar prevention accounting for another $12 billion.^31^ Commercially available dressings with regenerative capabilities include silver‐impregnated dressings, which are used extensively to prevent infections in the wound (e.g., Acticoat [Smith and Nephew], Fibracol [Johnson and Johnson, and Silvasorb [Medline]). Acellular grafts such as Alloderm (LifeCell), GraftJacket (KCI), Integra (Integra), and Biobrane (Smith and Nephew), and cellular grafts such as Dermagraft (Organogenesis), Epicel (Genzyme), and Recell (Avita) are also used.^32^ Traditional wound dressings are mainly designed to keep the injury site sealed and protected, and one of the main drawbacks is that they become attached to newly grown granulations and cause pain when removed.^33^ Recent advancements in wound healing technologies, such as multifunctional smart bandages, hold the promise of improving this outcome. New smart bandages are emerging that accurately measure the levels of important markers, such as pH,^34^, ^35^ temperature,^36^, ^37^ sweat/moisture,^38^, ^39^, ^40^, ^41^ oxygen,^42^, ^43^ hemodynamic and metabolic biomarkers,^44^, ^45^ periphery artery motion,^46^ blood pressure,^47^, ^48^ and electrical tissue properties.^49^

### 2.1 Monitoring of wound healing: Wearable pH, temperature, and moisture sensors

The integration of biosensors with a fully miniaturized wireless telemetry system enables the display of real‐time data for unobtrusive monitoring during the wound healing process. The pH value within the wound indirectly and directly influences all biochemical reactions taking place as the wound heals. Different pH ranges are required for certain distinct phases of wound healing, and the pH has proven to be a potent influential factor for the healing process.^50^ The skin's natural pH is more acidic (4–6.5) and increases during healthy wound healing (5.5–6.5). However, in chronic nonhealing wounds, the pH tends to be more alkaline and above 6.5.^51^, ^52^, ^53^ Infected wounds are either more acidic or alkaline, depending on the type of invading bacterial species.^54^, ^55^ Numerous pH sensors are emerging that allow for continuous monitoring of the wound environment's pH, which can serve as an alert to potential infection and nonhealing wounds. Colorimetric sensors are robust, easy to use and can be utilized without integrated electronics. Electrochemical pH sensors usually utilize potentiometric measurements, conducting polymers, pH sensors, metal oxide, ion‐selective electrodes, or ion‐selective field effect transistors.^56^, ^57^ A study in 2017,^37^ presented a low‐cost stretchable sensor, developed by spraying conductive inks and polymers onto a silicone substrate which was then coated with a conductive polyaniline conductive filler, binding material, and a pH‐sensitive membrane. Results demonstrated that the stretchable sensor displayed an optimal trace width of 0.3 mm could withstand elongations of up to 135% and was robust to more than 12,000 stretch‐and‐release cycles at 20% strain. The pH sensor displayed a linear sensitivity of −53 mV/pH with stable performance in the physiological range of pH 4–10. The integrated bandage exhibited Nernstian responses to cyclic changes in the environmental pH. Similar to pH sensors, flexible colorimetric and electrochemical temperature sensors have been widely used to monitor cutaneous healing. Many enzymatic reactions are temperature dependent and in particular, temperature is related to the inflammation and infection states of wounds.^58^ Research shows that a temperature difference between a chronically infected wound and normal tissue has a specific elevated thermal gradient range of 3–4°C. In wounds where the infection cleared but “healing” inflammation persisted, a thermal gradient of less than +2°C is reported.^59^, ^60^ In normal skin, a thermal gradient of ±1°C is observed.^61^ Thus, monitoring wound skin temperature may be a useful indicator of infection and treatment status. Microfabricated arrays of temperature sensors able to form a conformal contact with the skin have been used to map temperature distribution across wounds.^36^, ^37^, ^62^ Other examples of conformal on‐skin sensors include platinum nanofiber networks,^63^ reduced graphene oxide/polyurethane fiber elastomers,^64^ and gold‐coated conductive nanomeshes.^65^ Numerous methods and thermal‐sensitive materials have been used such as, conductive polymer ink (e.g., poly(3,4‐ethylenedioxythiophene) polystyrene sulfonate^66^, ^67^), and carbon‐based nanomaterials (e.g., a carbon nanotube forest^68^ and a reduced graphene oxide hydrogel^69^). Temperature sensors have also been designed to be transparent, by using materials such as liquid crystals,^70^ ionogels,^71^ and hydrogels.^72^ More than 40% of wound dressing changes occur before the optimal time,^73^ which cause unnecessary increases in cost and patient pain. The moisture level at a wound bed can be a good indicator for determining optimal intervals for wound dressing changes. A sufficiently moist wound bed facilitates wound edge contraction by enabling growth factors and numerous cell types, including epithelial cells, to migrate.^74^ However, overly moist wounds resulting from excessive exudate weeping hampers proliferative phase wound healing and thwarts inflammation reduction. To this end, the use of electrochemical sensors is emerging that measure wound moisture. When integrated with wound dressings, the electrochemical sensors can provide valuable information about optimal dressing change intervals and if excessive moisture is present—without the need to first remove the dressing. A leading moisture sensor example by WoundSense™, based on original work by Milne et al.,^38^ detects moisture content at the wound bed via low‐current electrical impedance. Here, the electrodes consist of silver chloride electrode printed on a flexible, biocompatible polymer, and a corresponding meter within the dressing is used to indicate whether the dressing is very dry, very wet, or of favorable conditions for healing. In another example, composites of carbon–zinc and carbon–manganese dioxide could correlate moisture level with the absorbing capacity of the dressing thereby informing the optimal dressing change interval.

### 2.2 Wound healing augmentation

**FIGURE 3:**
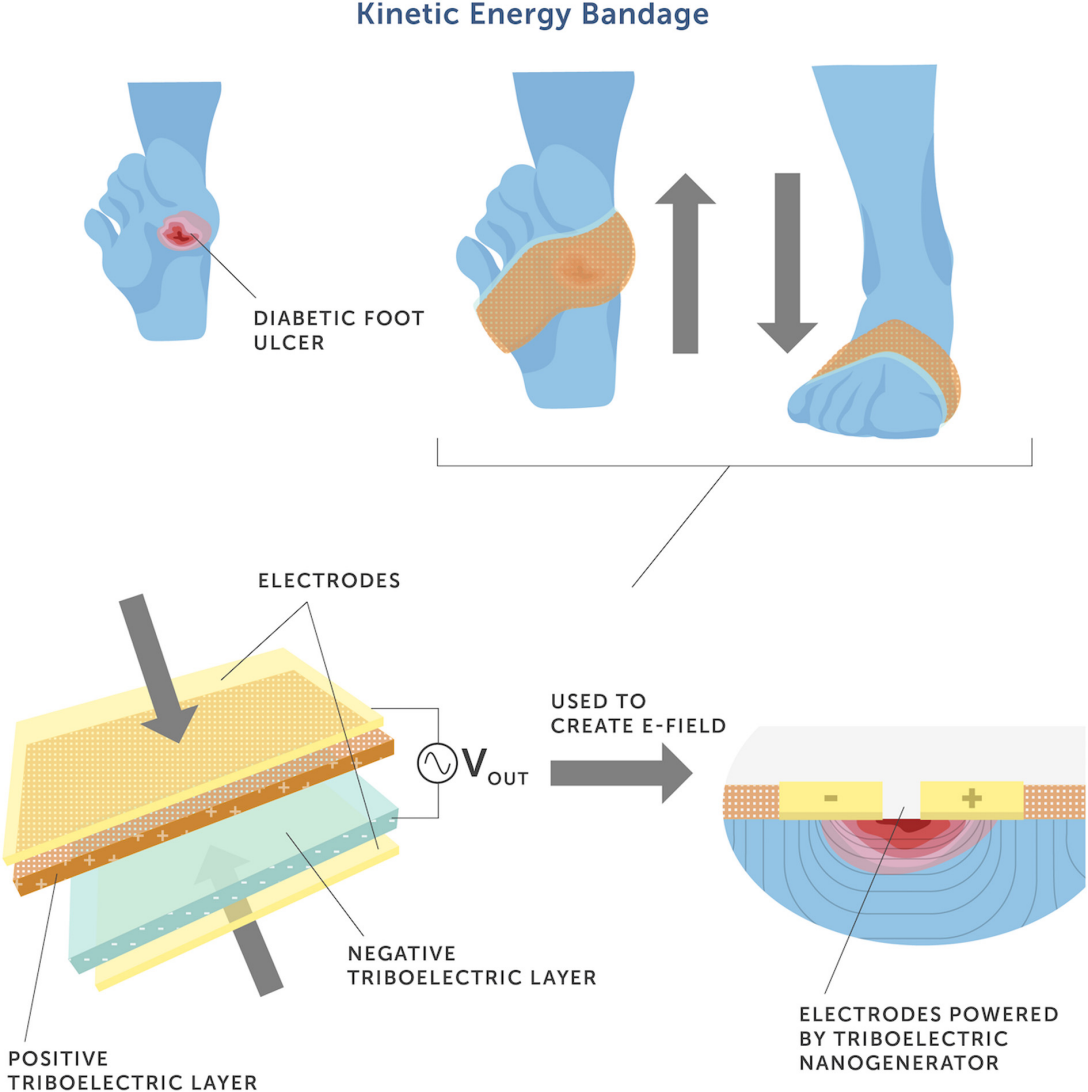
A schematic demonstrating the potential use of a kinetic bandage for patients with a nonhealing diabetic foot ulcer. An alternating discrete electric field is generated by a wearable nanogenerator by converting mechanical compression during walking into electricity.

In addition to the capabilities of the smart bandage in monitoring wound healing, more recent studies seek to develop devices capable of actively augmenting wound healing. In 1857, Dubois‐Reymond first observed that human skin is electrically active^75^ and more recent studies support that changes in electrical parameters in the wound microenvironment modulate the migration and function of many host cells and can improve wound healing.^76^, ^77^, ^78^ The continuing development of sensors, nanogenerators, automated drug delivery systems, and the employment of electrochemistry principles to the wound site offer immense possibilities for wireless smart bandage applications. Nanogenerators open a route for generating periodic biphasic electric pulses via electro‐mechanical or electro‐thermal transduction stemming from body or muscle movement.^79^, ^80^, ^81^ This capability makes nanogenerators a candidate for producing electrical stimulations that are self‐sustainable and within a biologically responsive range.^54^ Using this technology, a self‐activated electrotherapy bandage was developed that was able to convert kinetic energy generated by chest movements during breathing, into biphasic electric pulses.^54^ This self‐powered electric‐dressing device consisted of two parts: the biomechanical energy conversion part (i.e., the nanogenerator) and the dressing electrodes. The nanogenerator was constructed from polytetrafluoroethylene sandwiched between two copper electrodes and laminated on a polyethylene terephthalate (PET) film. The device was designed to drive charge flow towards or away from the two wound dressing electrodes, inducing an electrical potential in the area between the electrodes (i.e., across the wound bed). During normal activity, breathing generated a peak‐to‐peak voltage amplitude of 2.2 V at a rate of 110/min. Preliminary results up to 72 h showed no toxicity to host cells suggesting the potential safe application onto wound sites. Further in vitro analyses demonstrated that when fibroblasts, the key cells involved in wound healing, were exposed to a 2 V/cm electric field at a frequency of 1 Hz this facilitated fibroblast viability, proliferation, and transdifferentation into myofibroblasts; cells that provide a contraction force necessary for wound healing. These results support the role of electrical stimulation in regulating the cells involved in the highly coordinated biological process of wound healing. In vivo analyses following the application of an ~250 V/m electric field to full‐thickness skin wounds on the back of rats increased cell migration into the dermal wound bed, which resulted in accelerated wound healing (3 days to heal vs. 12 days in control animals). Overall, the study demonstrated that the dressing electrodes generated an electric field able to penetrate the dermis potentially strengthening the existing endogenous electric field, thereby significantly enhancing wound healing (Figure 3).

Cutaneous wound healing can be severely impaired by biofilm infection through disrupting epithelial barrier function.^82^ Despite evidence supporting a role of weak electrical fields (±12 V/cm with a low current density ± 2.1 mA/cm^2^) having antibiofilm properties,^83^ few in vivo studies have addressed the contribution if any, in preventing wound infections. In 2019, Barki et al.^84^ developed a novel wireless “electroceutical” dressing (WED) as an alternative, and nonpharmacological method to improve healing by mitigating polymicrobial biofilm infection within chronic wounds. The goal of this study was to test whether wounds complicated by infection, could be rescued by treatment using a WED in a porcine model. The textile‐based disposable bioelectric dressing was composed of a lightweight, single layer polyester dressing consisting of a polyester substrate containing elemental Ag (0.9 mg/cm^2^) and Zn (0.3 mg/cm^2^) bound to the surface in a dot matrix pattern, creating multiple micro batteries. In the presence of a conductive moist wound environment, low‐level microcurrents (2–10 μA) were generated between the Ag and Zn microcells at the device surface, generating an electric field that exerted antibiofilm properties. The dressings were changed twice a week for up to 56 days postinoculation. Results showed that the WED prevented and disrupted wound biofilm aggregates and accelerated functional wound closure. To determine the driving mechanism behind a reduction in biofilm formation, several skin barrier proteins were screened. E‐cadherin emerged as the single candidate that was repressed following biofilm infection by mechanisms that were sensitive to WED. Indeed, E‐Cadherin adhesion molecules are key determinants of morphogenesis and tissue architecture and E‐Cadherin is essential for in vivo epidermal barrier function.^85^ Promising preliminary results have also been demonstrated in patients.^86^

Technological advances are also being used to explore smart bandages as automated drug delivery systems and diagnostic tools.^49^ The ability to detect and monitor pathogenic infections, then release antibacterial drugs directly at the wound site could revolutionize expedient patient care. A study by Mirani et al.^32^ demonstrated a unique and advanced multifunctional hydrogel‐based dressing (GelDerm) composed of two parts: a pH‐sensitive colorimetric component and a drug‐eluting component. First, the integrated wound dressing colorimetrically measured changes in pH at the wound bed, which served as an indicator of bacterial infection. Second, and following visual detection of an infection, the user instructs the dressing to release antibiotics directly at the wound site. Researchers were able to map the pH of the wound bed using an array of bioprinted porous sensors (12 mm × 12 mm) composed of 3D‐printed color‐changing alginate fibers. Following the onset of infection, color changes were spatial visualized imaged, processed and analyzed with less than ±5% error using an in‐house smart‐phone application (iDerm) system. Similarly, drug eluting alginate scaffolds were fabricated by 3D‐printing gentamicin‐loaded alginate fibers. Pig skins were inoculated with *Pseudomonas aeruginosa*, the dressing was applied, and clear color changes were observed in the infected samples. Tests confirmed the hydrogel was able to release a high dose of gentamicin at the wound site, and that a dose of 3 mg/ml^−1^ was needed to eradicate the bacteria. A future aim of this technology is to develop the system to detect more specific bacterial markers, which would aid in sepsis diagnosis.

Wound healing is dynamic, with at least four distinct phases. Thus, the ability to administer therapies expeditiously and at the right time, could further enhance the ability of a smart bandage to augment wound healing.^87^ This would require a combined approach of real‐time monitoring with on‐demand drug delivery using a more sophisticated closed‐loop approach. Here, Mostafalu et al.^87^ developed a novel, closed‐loop stimuli‐responsive flexible bandage. The multicomponent bandage consisted of sensors (pH and temperature), a microheater, and thermo‐responsive drug carriers (poly(*N*‐isopropylacrylamide) [PNIPAM]‐based particles) embedded within an alginate hydrogel patch. A wireless microcontroller was used to process the data measured by the sensors and to automatically program the drug release protocol for individualized treatment. The integrated bandage was less than 3 mm thick and changes in pH were interpreted as an indication of bacterial infection. Potentiometric pH sensors composed of carbon/polyaniline operated under the principle of protonation and deprotonation of the electrode in an acid and basic environment. The charge accumulation resulted in a voltage output measurement that was used to determine the pH; demonstrating a linear response and sensitivity of −50 mV pH^−1^. Thermo‐responsive drug carriers embedded within the hydrogel were programmed for release in response to temperature variation, where stimulation above a threshold value activated the integrated heater to release antibiotics. Thermo‐responsive PNIPAM particles, approximately 300 μm in diameter, were fabricated using a microfluidic flow technique. Particles were immersed in a solution of the antibiotic cefazolin prior to encapsulation within a thin rectangular patch of alginate. Remarkably, the rate of antibiotic release from the dressing was controlled by varying the temperature conditions. Higher temperatures led to a faster drug release rate, a reduced temperature significantly slowed the rate, while the reapplication of higher temperatures restored faster drug release from the dressing. Similar to the automated and controlled release of the antibiotic via the temperature sensors, release of cefazolin was also achieved via changes in pH. Growth of *Staphylococcus aureus* within a bioreactor reduced the pH to 6.5, which automatically activated cefazolin release, and subsequently raised the pH to 7.2, deactivating further drug release. The smart bandage reduced the growth and viability of *S. aureus* from 100% in the control group, to less than 10% in the treated group. Readout from the sensor and stimulation of the thermo‐responsive drug were achieved remotely.

The ability to promote healthy wound healing in the elderly combined with a smart system able to diagnose and treat infections directly at the site of injury should they arise, holds considerable promise. Point‐of‐care devices coupled with telehealth or digital health offers the potential to transform healthcare toward home‐based personal medicine, reducing healthcare costs, time for diagnosis, decreasing wound dressing exchange, and the unnecessary administration of antibiotics and other drugs. This would directly support the elderly in the management of chronic and acute injuries caused by trauma, surgery, or diabetes.

## 3 THE COCHLEAR IMPLANT

Clinically meaningful hearing loss is highly prevalent and a major public health issue that affects nearly two thirds of the elderly population.^88^ Currently, over 16 million adults in the United States over 70 years of age suffer from hearing loss and this number is anticipated to almost double by 2060 given the aging population.^89^, ^90^ Oldest‐old individuals (>85 years) display a higher prevalence of hearing loss (41% in at least 1 ear, 38% bilateral) and more severe levels of loss than those who are younger.^88^ Thus, it is anticipated that by 2060, the number of people with a moderate or greater hearing loss will exceed the number of people who have a mild loss today.^88^ Furthermore, recent studies have demonstrated the association between hearing loss and poorer health outcomes,^91^ including an increased risk for cognitive impairment,^92^ accelerated cognitive decline over time,^93^ and dementia.^91^ Although some studies have shown no association.^94^

The cochlear implant (CI), introduced in 1957, is the oldest type of neuroprosthetic. The CI bypasses damaged neural regions within the ear by translating sound from the environment through direct electrical stimulation of the auditory nerve, within the cochlea. The implant's receiving coil, magnet, internal processor/receiver, and electrodes, which are housed along an electrode array carrier, is surgically placed under the skin. These internal components rest on the surface of the skull and the electrode array is placed within the cochlea where it receives sound information via transcutaneous transmission of the signal to the antenna of the internal receiver coil.^95^ The external head‐set components, microphone, processing unit, connecting cables, and transmitter coil, analyze, process and transmit sound information to the internally implanted device. Briefly, sounds are picked up by the microphone, the speech processor converts the sound into a coded electrical signal. The coded signal travels to the external transmitter, which sends the signal to the internal receiver where the information is decoded, delivering electrical stimulation to the implanted electrodes. Over the last three decades, CIs have evolved from an experimental procedure to represent the standard of patient care. Presently, the CI prosthesis aids over 200,000 deaf children and adults around the globe by partially restoring hearing. More than 150,000 of these individuals are elderly.^96^

The rapid advancement in materials science, electronics and coding strategies has led to highly sophisticated electrode arrays, implanted electronics, and sound processors that can deliver patterned auditory information at rapid rates to surviving auditory neurons within the cochlea.^97^ Today, most profoundly deaf individuals who use a CI are able to detect speech sounds within a normal range of hearing (i.e., 25 db HL).^98^ However, and despite their remarkable success at restoring speech perception, several limitations remain. Current CI users have limited pitch perception,^99^ frequency resolution,^100^ and dynamic range.^101^ These challenges in extracting basic sound properties translate into limitations that include the ability of the user to understand speech in noisy environments,^102^ locate sound sources,^103^ and enjoy music.^104^ As a result, medical device manufacturers are focusing on developing innovative CIs. In 1976, 3 M CI systems used only a single electrode and was referred to as a single‐channel device. Current systems use between 12 and 24 multichannel electrodes and offer various lengths, sizes, and configurations.^105^ Early multichannel CI systems used straight yet flexible electrode arrays, while in contrast, current arrays are precoiled, allowing the electrodes to be placed closer to the center of the modiolus where spiral ganglion cells are located.^98^ These advancements have led to amplified precision in simulating the cochlea by more accurately inputting the signals and delivering a clearer sound.^106^ Additionally, contemporary arrays are designed to be less damaging to the delicate structures in the cochlea during surgical placement, thereby preserving residual hearing. In recent years, the sound processors that feed signals to CIs have also undergone many developments and manufacturers such as Cochlear and MED‐EL now have seven generations of implantable components and nine generations of sound processors. In 2016, Cochlear introduced the Kanso® Sound Processor, which combines dual‐microphone technologies with the Auditory Scene Classifier to automatically adjust the sound processor to different listening environments. Here, the audio signal is analyzed and captured by the processor microphone and multiple relevant acoustic features are extracted, including spectral shape, overall level, modulation rate and depth, and tonal information.^107^ This is followed by environmental classification, where the extracted acoustic cues are compared to environmental templates from within a fixed set of acoustic scenes including quiet, speech in quiet, speech in noise, noise, wind, and music. The algorithm employed is the best match between the user's environmental acoustic conditions and the stored classified environments. The Nucleus 6 also provides adaptive sound processing qualities and performs acoustic scene classification, adapts for directionality, and automatically adjusts the sensitivity and input signal processing to maximize performance in noise.^107^ This resulted in a significant mean improvement in sentence recognition when compared to an older design. Further, CIs can now connect directly to the user's smartphone, television, and other Bluetooth‐enabled devices.

The current frontiers involve strategies to preserve residual acoustic hearing and the development of algorithms that combine electrical and acoustic hearing.^108^ As hearing loss develops, individuals often first lose their high‐frequency hearing (2000–20,000 Hz) and with further progression, the loss may also involve lower frequency (20–200 Hz), acoustic hearing. For CI users with useful residual acoustic hearing, combining electrical CI stimulation with acoustic stimulation (electro‐acoustic or hybrid stimulation) has been shown to preserve residual hearing and improve performance.^109^, ^110^, ^111^ Hybrid stimulation involves inserting a shortened, minimally traumatic, CI electrode array into the basal cochlea to stimulate the high‐frequency region, while a conventional acoustic hearing aid is used to deliver amplified acoustic and low frequencies. This combination augments the quality of sound and speech, performance in background noise and improved sound localization compared with hearing aids and CIs alone.^110^, ^112^ However, despite the relative high rates of success, postoperative acoustic hearing outcomes remain unpredictable and a subset of patients suffer from partial to total loss of acoustic hearing months to years following surgery.^105^ Low frequencies contribute to speech perception and individuals with impaired low‐frequency hearing are usually only able to detect vowels, but few or no consonants; missing information such as pitch, temporal fine structure and dynamic changes in intensity. As such there is difficulty with word understanding and hearing in noise, particularly in environments such as classrooms, cafes, and busy workplaces.^113^ Recent work has demonstrated that sensory substitution via tactile presentation of this low‐frequency sound information could improve speech‐in‐noise performance for CI users with impaired low‐frequency hearing. As such, electro‐haptic stimulation has recently emerged as an alternative approach to improve CI outcomes. In normal‐hearing individuals, the origin of a sound is determined by the intensity and arrival time of sounds as well as by the direction‐dependent spectral filtering of sounds by the pinnae.^114^ Due to the poor spectral resolution of CIs and the fact that CI microphones are typically mounted behind the ears, CI users are limited to this important spatial information and users struggle to discriminate sounds coming from different locations.^115^ In 2019 and 2020, Fletcher et al.^114^, ^116^, ^117^ provided missing spatial hearing cues through haptic stimulation of the wrists. In‐house signal processing was used to convert an audio signal to a tactile signal (50–230 Hz). The authors were able to extract their tactile signal from speech‐in‐noise and present it to the distal phalanx of the index finger, and in later studies, to the wrist. The tactile signal was presented through a HVLab tactile vibrometer with a 10 mm contacting probe and controlled using custom MATLAB scripts. Training consisted of 20 min of exposure to speech‐in‐noise and concurrent vibro‐tactile stimulation over the course of two 1‐h sessions. Electro‐haptic stimulation was found to improve the intelligibility of speech in multitalker noise for normal‐hearing indivduals,^118^ improve speech recognition,^117^, ^119^ and participants were able to more effectively integrate spatial information, thereby dramatically improving sound localization.^114^ These studies suggest an important step toward providing a noninvasive and inexpensive approach to improving outcomes for CI users. Further research also seeks to develop optical CIs, although much work is required to optimize this technology. Briefly, infrared light has been shown to offer higher spatial selectively and greater sound resolution, thereby augmenting activation of the surviving neurons in the cochlea.^120^

Age‐related hearing loss may be a late‐life modifiable risk factor for adverse health outcomes in the elderly. Despite the high prevalence of hearing loss, and significant advancements in CIs (including robotic‐assisted CI placement^121^, ^122^), treatment remains vastly underutilized, with fewer than 15% of hearing impaired adults over 50 years in the United States using a hearing aid.^123^, ^124^ The lack of CI use may include limited awareness and understanding of the impact of hearing loss or the treatment options that are available, high costs, stigma, and challenges with technology and its complexity.^124^ Further there are drawbacks of the current CI strategies including discomfort, noise feedback, recurrent inflammation, and exacerbation of ear canal infections.^110^ Of note, these challenges have inspired the development of intracochlear drug releasing electrode carriers (e.g., glucocorticosteroids, zwitterionic hydrogels, antiapoptotic and anti‐inflammatory substances, and neurotrophins) in combination with CIs. Previous review articles comprehensively cover intracochlear drug release.^105^, ^125^ Due to the expected rapid growth in the number of elderly individuals with hearing loss over the next several decades, there is an increased need for affordable interventions, both preventative and therapeutic. If the advancements in CI technology offer even a small beneficial effect of hearing aid use, this would have significant implications for public health given that traditional hearing aids are readily available and are currently not utilized by nearly 23 million older adults with hearing loss.^124^

## 4 AI IN MEDICINE

More than 85% of elderly Americans have at least one chronic disease and 65% have at least two.^126^ Cardiovascular diseases, cancers, chronic respiratory disease, and diabetes impose a disproportionate impact on the overall disease burden in the elderly population.^127^ Over the next few decades as the global population rapidly progresses toward a super‐aging society, meeting the increasingly long‐term quality of care needs is forecast to present a major healthcare challenge. Traditional healthcare models based on in‐person monitoring are likely unsustainable on their own in supporting the projected increase in patient demands, which will require higher levels of personal attention, assistance, and care.^128^ This challenge will require healthcare providers, and even countries to seek and invest in new cost‐effective models able to support these needs at the scale of big data. Recent advancements with AI coupled with machine learning (ML; AI‐ML) in the healthcare arena is likely to play a pivotal role in reshaping care services for older adults through improving, (1) diagnostic accuracy, (2) chronic care management, and (3) health care delivery systems. AI‐ML is a promising technology to complement the current care provision, improve overall quality of care, and enhance older adults' ability to live safely in their desired settings.

**FIGURE 4:**
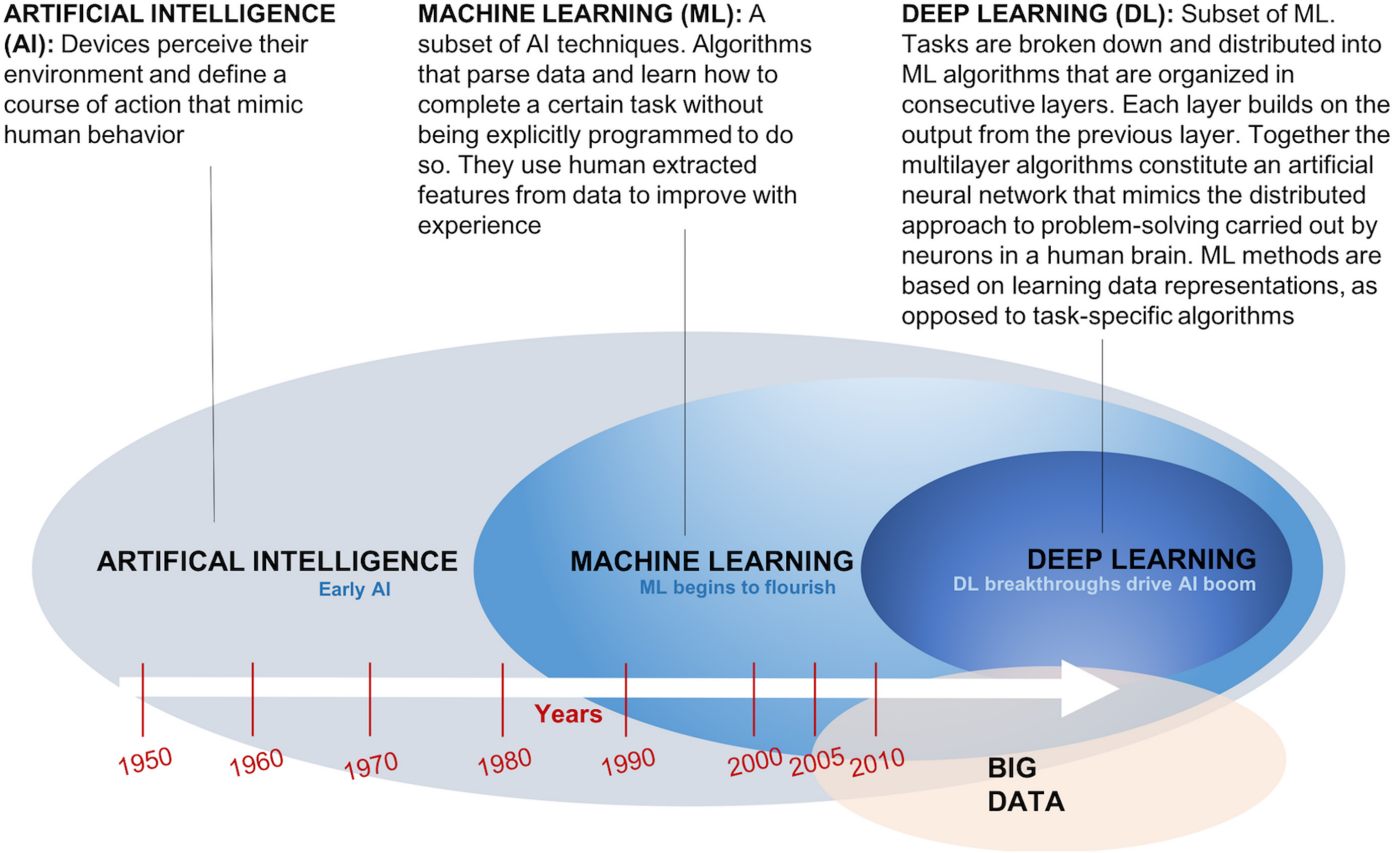
A schematic of the development of artificial intelligence, machine learning, deep learning, and big data over recent years

**FIGURE 5:**
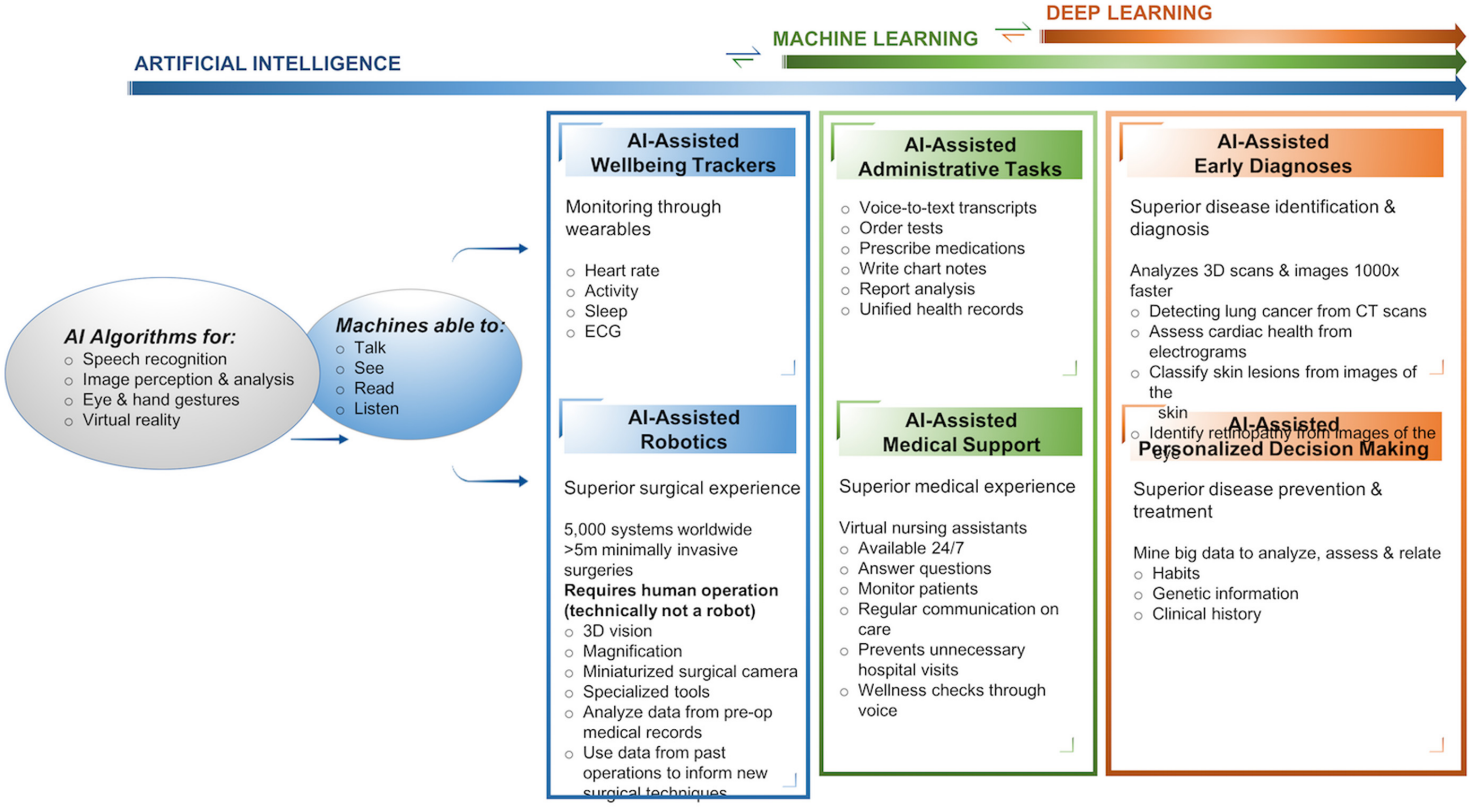
A schematic showing the progression of artificial intelligence (AI), AI‐ML, and the use and potential use of deep learning within medicine. AI is forecast to provide a superior surgical and medical support experience, improve the time and accuracy of administrative tasks as well as offer a superior level of disease identification, early diagnoses, and disease prevention and treatment.

AI‐ML computing technologies are now able to perform many tasks that were once considered solely the domain of humans. Through mimicking human intelligence, AI is endowed with processes such as recognizing, learning, adapting, predicting, and deciding. These processes converge to produce machines that approximate the skills of talking, seeing, reading, listen, and even learning through watching videos. A resurgence in AI has recently come about due to advancements in ML brought about through advances in analytical computational power paired with enormous amounts of data that are being generated (automobiles, health monitoring, digitization of images, and other medical information) (Figure 4). Deep learning, a subset of ML, uses statistical techniques to empower the computer to make predictions and decisions on their own and based on past data, allowing the programs to perform tasks progressively better through iterative experiences.^129^ Healthcare and medicine stand to benefit immensely as these optimization algorithms can continually collect and analyze a vast amount of data from longitudinal observation, identify and categorize patterns, and use predictive analytics to assess risk level and make behavioral or care recommendations^128^ (Figure 5). For example, AI‐ML‐enabled blood pressure or electrocardiogram monitors may help to predict or diagnose various health concerns common to the elderly (e.g., hypertension and atrial fibrillation). Refined AI‐ML home‐health monitoring systems, such as computer vision analytics, can detect and dissect daily activities such as standing or walking and then iteratively learn the expected levels for an older individual (e.g., walking speed, total daily activity, time out of home, and ability when getting out of bed).^130^, ^131^, ^132^ This is of importance as the parameters measured during activities of daily living including, bathing, toileting and cooking are proven indicators of the cognitive and physical capabilities of the elderly.^133^ The AI‐ML system can collect complex streams of data in real‐time, longitudinally track movements, analyze the input, and identify any unusual changes that may suggest the onset of cognitive or functional decline. Using predictive reasoning, the automated analytic system can then intervene by sounding an alert or offering behavioral suggestions in response to what may be subtle declines in function, which a human may otherwise miss.^128^, ^134^ As a result, these systems enable a better understanding of the dynamic nature of an older adult's disease progression or functioning^128^, ^135^ and can potentially facilitate timely access to appropriate healthcare and prevent serious injuries.^136^, ^137^

AI in medicine is considered either virtual or physical. Virtual AI‐ML tasks range from applications such as guidance in treatment decisions, to electronic health record systems, analysis and detecting mistakes in treatment and workflow inefficiencies to avoid unnecessary hospitalizations.^138^ Physical AI‐ML consists of robots assisting in performing surgeries, and in intelligent prostheses for use following disability.^139^ Some of the greatest successes of deep learning have been in the field of computer vision. Deep learning models have achieved physician‐level accuracy in a variety of complex diagnostics. These include identifying moles from melanomas,^140^, ^141^ detecting diabetic retinopathy or predicting cardiovascular risk from images of the retinal fundus,^141^, ^142^ the diagnosis of retinal disease,^143^ computer‐aided diagnosis of breast lesions^144^, ^145^ and in the detection of cancer.^142^, ^146^ Beyond this, in 2018, an algorithm called deep learning‐based automatic detection (DLAD) was successfully developed by researchers in Seoul and is able to analyze chest radiographs and detect abnormal cell growth and malignant pulmonary nodules, outperforming the detection by 94% of the doctors included in the study.^147^ Another deep learning example was developed by Google AI Healthcare who created lymph node assistant (LYNA). This system was able to successfully analyze histology slides prepared from lymph node biopsies and identify metastatic breast cancer tumors with a 99% accuracy.^148^ The authors reported that LYNA was able to recognize suspicious regions within the tissue that were undistinguishable to the human eye and halved the average slide review time. A limitation recently suggested, however, is the lack of clinical context as deep learning methods will constrain the diagnosis to be performed using just the images at hand.^129^ Using this more focused approach introduces difficulties for a human reader, who in real‐world clinical settings has access to both the medical imagery and supplemental data, including the patient history and health records, additional tests and patient testimony. Conceivably, this emphasizes the need for a combined and collaborative approach where AI‐ML assists the clinician in providing accurate diagnoses leading to recommended treatment, therefore improving the health of the elderly.

Comorbidities and frailty represent well‐known problems during and after surgery in elderly patients. The oldest‐old population are less able to tolerate the stress of medical illness, hospitalization and immobility, making surgery a substantial problem.^149^, ^150^ This is of particular concern to surgical oncologists as the number of cancer diagnoses is forecast to increase with the aging population, and in this aged population, cancer often occurs as an advanced stage of the disease.^151^, ^152^ Recent studies demonstrate the feasibility of surgical treatment in the elderly for several types of cancer including, sarcomas,^153^ ovarian cancer,^154^ colorectal cancer,^155^ and pulmonary cancer.^156^ Minimally invasive surgery offers many advantages including earlier postoperative mobilization, reduced blood loss, lower morbidity and less postoperative pain, and has been shown to be better tolerated than open surgery in the elderly.^155^, ^157^, ^158^ Arguably, the most commonly recognized application of AI is in the use of robots. The eponymous da Vinci Surgical System has become one of the most commonly used robotic technologies in the field of minimally invasive surgeries.^159^ The machine is typically used for prostatectomies and gynecological procedures, in oncologic surgery, and has recently gained usage in cardiac valve repair,^160^, ^161^, ^162^, ^163^, ^164^, ^165^, ^166^, ^167^ thereby allowing more people to benefit from the minimally invasive surgical approach. As of 2020, there are nearly five thousand da Vinci Systems worldwide that have successfully performed well over 5 million surgeries. As this machine currently requires human operation to perform human operation, it is, by definition, not independent AI. The procedures are performed by surgeons who control the system, and as such allow the operator to pass on the limits of human ability or repeatability. Its features include 3D vision, magnification, a miniaturized surgical camera, specialized tools, and robotic and computer assistance.^168^ Through using computer vision models, deep learning can enhance robotic assisted surgery by perceiving and reconstructing surgical environments and through learning from a surgeons' physical motions.^129^, ^169^ Deep imitation learning, recurrent neural networks, and trajectory transfer algorithms are able to fully automate certain teleoperated manipulation tasks of the surgical procedure.^170^ This supports the automation and speed of highly repetitive and time‐sensitive surgical tasks, such as suturing and knot‐tying.^129^, ^170^ This type of research will undoubtedly have a profound impact on further improving patient care, especially the oldest‐old, potentially improving surgical accuracy and reducing surgery time, while decreasing stress and improving postoperative recovery.

In summary, advantages in AI‐ML and neural networks include an increase in diagnostic and therapeutic efficacy, and a reduction in medical error while simultaneously decreasing administrative workload and cost. However, skeptics recommend proceeding with caution as AI‐ML‐augmented practice may also reduce job opportunities, and remove the interaction and communication offered by human touch, empathy and emotional intelligence. Other concerns and risks associated with AI‐ML and its use in the elderly population include the depersonalization of care through algorithm‐based standardization, the discrimination of minority groups through generalization, the dehumanization of the care relationship through automization, and disciplining users through monitoring and surveillance.^171^ The rise of AI‐ML and the pursuit of finding a mutually beneficial and collaborative balance for older adults and their caregivers will be essential.

## 5 MEDICAL MICRON AND NANOROBOTS

Morbidity is broadly defined as the state of being unhealthy, sick, diseased or having genetic or anatomic pathologies or injuries; many conditions that are common in the elderly population. The engineering of bioinspired and molecularly precise micron and nanorobotic structures holds considerable promise for advancing medical diagnosis and treatment, thereby reducing morbidity. This is due to their unique ability to move and perform complex tasks at small scales (300 nm to 300 μm), thus permitting intervention in biological processes at the molecular level.^172^ By definition, this offers the potential to target treatment toward individual organs, tissues, cells, and even intracellular components. In essence, medical robots hold the promise of a nanomedical engineered solution able to cure disease, reverse physical trauma and promote individual cell repair, thus regulating human aging.^173^ As a consequence, the global market for nanodevices and nanomachines is expected to grow from $736 million in 2018, to $1.3 billion in 2023, and then to $2.7 billion in 2028 (a CAGR of 16.0% from 2023 to 2028).^174^ Emerging areas of research aim to build nanoscale molecular parts such as gears, motors, pumps, sensors, bearings and ratchets; structures capable of converting power sources into kinetic energy. Remarkably, each nanopart may comprise a few thousand precisely placed atoms. To facilitate locomotion, biohybrid systems integrate synthetic nanostructures with motile microorganisms used as the engine.^175^, ^176^ Chemically powered micron or nanorobots use asymmetric catalytic engines to selectively convert chemical fuels into locomotion.^177^, ^178^ Physically powered nanorobots convert external energy inputs (e.g., magnetic, ultrasound, or light fields) into translational motion based on engine geometry and material designs.^179^, ^180^

DNA‐based nanorobots able to sense, transport, actuate and exert functions when exposed to external signals are being investigated as one avenue for the delivery of therapeutic cargoes in targeted drug delivery (TDD).^181^, ^182^ DNA has been shown to be an excellent substrate for the design and construction of mechanical nanorobots. Interactions of complementary base pairs and extensive knowledge of its chemistry allow folding of DNA into specific 2D and 3D shapes in a process known as DNA origami.^183^, ^184^ This technique produces nanostructures of a controlled size, shape and spatial orientation, generating functional platforms for broad biological application.^185^, ^186^, ^187^ Further, the outside surface of the DNA nanorobot can be functionalized with, for example, a DNA aptamer of known high binding affinity toward a target protein. Protein binding subsequently produces a trigger mechanism that instigates release of the cargo at the tissue or cell site.^188^ A study performed by Li et al.^189^ described the development of an autonomous DNA nanorobotic system based on a self‐assembled origami nanotube. The outer surface of the nanorobots was functionalized with a DNA aptamer with a binding affinity for nucleolin, a protein specifically expressed on tumor‐associated endothelial cells. The nanorobot also carried an internal payload of the blood coagulation protease, thrombin. The nucleolin‐targeting aptamer served both as a selective targeting domain and as a molecular trigger for the mechanical opening of the DNA nanorobot, where the released thrombin activated coagulation at the tumor site. Remarkably, the controlled delivery of active thrombin led to vascular occlusion, nutrient and oxygen deprivation, and subsequent tumor necrosis. The DNA nanorobotic system consisted of several elements that were constructed sequentially. First, a rectangular 90 × 60 × 2 nm DNA origami sheet was prepared by assembling a M13 bacteriophage genome DNA strand and multiple staple strands. To load thrombin, capture strands with poly‐A sequences were attached at four predetermined locations on the surface of each sheet. To facilitate thrombin‐DNA conjugation, thiolated poly‐T oligonucleotides were tethered to the thrombin molecules using the crosslinking molecule sulfosuccinimidyl‐4‐[*N*‐maleimidomethyl] cyclohexane‐1‐carboxylate. On mixing, the poly‐A strands on the DNA sheet hybridized with the poly‐T strands tethered to the thrombin molecules, allowing the thrombin molecules to anchor onto the surface of the DNA sheet. The DNA‐thrombin sheet was then converted into a nanotube where the thrombin molecules were selectively attached to the inner tube surface (tubes were ~19 nm in diameter and 90 nm in length). DNA oligonucleotides with specific binding affinity for nucleolin were hybridized to the outer surface of the nanotube using designer fastener strands. The localization and efficacy of these thrombin‐carrying nanorobots were investigated in two different in vivo animal models. First, and following intravenous administration, the nanorobots were demonstrated to accumulate in MDA‐MB‐231 breast cancer tumors in mice where they triggered platelet aggregation and thrombosis. In contrast to the control animal group, tumors treated with thrombin‐carrying nanorobots displayed advanced tumor necrosis and decreased tumor growth, with no cytotoxic or adverse immunological effects noted. Second, and as the relative avascularity found within the core of a tumor presents a major therapeutic delivery challenge, nanorobotic efficacy was investigated in tumors with reduced vascularity. The thrombin‐carrying nanorobot was administered to mice with established and poorly vascularized SK‐OV3 ovarian cancer xenografts and results demonstrated prolonged animal survival. Although the inhibitory efficiency of the nanorobots was not as remarkable as observed with the vascularized tumors, this result nevertheless suggests that limited vascularity to the tumor did not completely inhibit therapeutic efficacy. Limitations of this system appeared to be reliance on systemic circulation as the main method of nanorobotic delivery to the tumor site, in addition to a lack of the propelling force needed for the nanorobots to penetrate further into the tumor and beyond their diffusion limits.^190^

Alongside the challenges created by avascularity, oxygen consumption by the rapidly proliferative tumor cells results in approximately <0.7% O_2_‐depleted tumor areas; referred to as hypoxic regions.^191^ To further improve TDD and the challenge of delivering therapies within the oxygen‐depleted hypoxic regions located deeper within the tumor mass, and beyond diffusion limits, the use of motile biohybrid nanorobots with magnetically stimuli‐responsive apparatuses have been investigated.^139^, ^192^ A species of Alphaproteobacteria called *Magnetococcus marinus* has been of special interest as it contains a chain of magnetic iron‐oxide nanocrystals.^193^ The MC‐1 (magnetic coccus #1) strain has been classified as an obligate microaerophile and as such, requires oxygen to survive but requires lower than atmospheric levels for optimal living; conditions similar to the environment within the hypoxic region of a tumor. Magnetotactic cocci are unique in that they persistently swim along local magnetic field lines and orientate themselves along the Earth's geomagnetic axis. In their natural environment, these bacteria are motile in either direction and use magnetotaxis in conjunction with aerotaxis, that is, magnetically assisted aerotaxis or magnetoaerotaxis, to migrate and maintain a position at their preferred low oxygen concentration.^194^, ^195^ The bacteria preferentially migrate to the oxic–anoxic transition zone, and through this attribute, may preferentially target a tumor between the angiogenic network (oxic) and the tumor necrotic (anoxic) zones. This presents a novel approach to advance drug targeting to the challenging hypoxic regions of a tumor.^196^, ^197^ An external magnetic stimulus is used to provide initial guidance of the nanorobots toward the tumor site, after which the innate swimming properties of the bacteria take over to deliver the therapeutics to the target tissue.^139^, ^197^ A study performed by Felfoul et al.^197^ demonstrated that 55% of magnetically guided *Magnetococcus* MC‐1 cells, each carrying ~70 covalently bound nanoliposomes containing drug payloads, entered the hypoxic regions of HCT116 colorectal xenografts after peritumoral injection in SCID Beige mice. MC‐1 cells preferentially localized in the hypoxic regions of the xenografts, as well as displayed a substantial increase in the targeting ratio and distribution in the tumoral volume following magnetic guidance. MC‐1 cells also demonstrated superior TDD when compared to passive diffusion methods that were achieved through standard administration. Exploiting the innate properties of these nanorobots when in abundant quantities, offers the potential to significantly advance and improve the therapeutic oncologic outcomes in both the younger and elderly population.^139^, ^197^

The application of micron and nanorobotics in medicine is vast. Not covered in this review but of note are chemically‐ and self‐propelled microrobots used as a novel approach to improve wound healing.^198^, ^199^ Further, microrobots with star‐shaped grippers have been used to excise tissue biopsy samples,^200^ and a magnetically actuated bacteria‐like microrobotic with a flagella and fluorescent tag, has been used to image the inside of the peritoneal cavity of a mouse in real time.^201^ Important advantages of medical micron and nanorobotics over present‐day approaches include improved speed of treatment. Mechanical nanorobotic therapeutic systems can reach their targets up to ~1000 times faster, and treatments that require ~10^5^ s (~days) for biological systems to complete may require only ~10^2^ s (~minutes) using nanorobotic systems.^202^ A second advantage is the improved control of treatment. Present‐day biotechnological approaches/devices/drugs are not programmable and cannot be easily switched on and off. The mechanical or electronic nanocomputer‐controlled nanorobotic approach may allow precise control of action, physical placement, timing, and molecular interaction.^203^, ^204^ Through the use of real‐time sensory feedback, a further advantage offered is verification of treatment to the physician. This technology could provide a summary of diagnostically or therapeutically relevant data describing what was found prior to treatment and what problems, if any, were encountered after treatment. Micron and nanorobotics may also offer minimal side effects and a more precise diagnosis. If successfully developed, and potentially via the administration of multiple micron and/or nanorobotic types, the future scope, benefit, and impact to the health and treatment of the elderly, especially, the oldest‐old, has the capacity to be enormous.^173^ However, and although their use is conceivable, clinical aspirations are still beyond the current capabilities of nanotechnology and bioengineering and significant limitations remain, including diffusion limits and biocompatibility requirements.

## 6 CONCLUSION

As the world's population ages, management of chronic disease will grow in importance. As life is extended, patients' health expectations rise, as today's elderly are already demonstrating their desire to remain active members of their communities, no longer satisfied with being shuttered in nursing homes after retirement. Improvement in accessibility access and efforts of sustaining activity further into late age has pressed technological development and medical standard of care. Our technological age has been answering the patient's call, and commercial enterprise continues to develop medical devices and applications, like those found in smart watches, to track personalized health outcomes. Now, Bio‐Implants, like smart bandages, the CI, the big data benefits of AI, machine learning, artificial neural networks, and nanorobotic drug delivery, may expand the modern physician‐scientist's medical bag, and in the process, help to extend healthy lives. Perhaps the novel medical technologies outlined in this review will lead to a new clinical definition of vital signs, where the measure of life is calculated to include a patient's quality of years and its quantity.

## AUTHOR CONTRIBUTIONS

**Albert Manero:** Conceptualization (equal); data curation (equal); methodology (equal); writing – original draft (equal); writing – review and editing (equal). **Hannah Prock‐Gibbs:** Data curation (equal); writing – original draft (equal). **Neel Shah:** Data curation (equal); writing – original draft (equal). **Deep Gandhi:** Data curation (equal); writing – original draft (equal). **Kaitlyn E. Crawford:** Conceptualization (equal); methodology (equal); visualization (equal); writing – review and editing (equal). **Melanie J. Coathup:** Conceptualization (lead); data curation (supporting); methodology (lead); project administration (lead); supervision (equal); writing – original draft (equal); writing – review and editing (equal).

## CONFLICT OF INTEREST

The authors have no conflicts of interest to declare.

### PEER REVIEW

The peer review history for this article is available at https://publons.com/publon/10.1002/btm2.10359.

## Data Availability

The data that support the findings of this study are available from the corresponding author upon reasonable request.
